# Metabolomic signatures associated with muscle dysfunction during the SIRIUS-21 space analog mission

**DOI:** 10.3389/fnetp.2026.1970105

**Published:** 2026-10-26

**Authors:** Adel B. Elmoselhi, Catherine Taylor, Vishwajeet Shankhwar, Andrew Blaber, Zeinab Ibrahim, Hamza M. Al-Hroub, Nelson C. Soares, Nandu Goswami

**Affiliations:** 1 College of Medicine, University of Sharjah, Sharjah, United Arab Emirates; 2 Research Institute of Medical & Health Sciences, University of Sharjah, Sharjah, United Arab Emirates; 3 Aerospace Physiology Laboratory, Simon Fraser University, Burnaby, BC, Canada; 4 Department of Electronics and Communication Engineering, Thapar Institute of Engineering & Technology, Patiala, Punjab, India; 5 Center for Space and Aviation Health, College of Medicine, Mohammed Bin Rashid University of Medicine and Health Sciences, Dubai, United Arab Emirates; 6 Center for Applied and Translational Genomics (CATG) Mohammed Bin Rashid University of Medicine and Health Sciences (MBRU), Dubai Health, Dubai, United Arab Emirates; 7 College of Medicine, Mohammed Bin Rashid University of Medicine and Health Sciences (MBRU), Dubai Health, Dubai, United Arab Emirates; 8 Laboratory of Proteomics, Department of Human Genetics, National Institute of Health Doutor Ricardo Jorge (INSA), Lisbon, Portugal; 9 Comprehensive Health Research Centre (CHRC), NOVA Medical School, University NOVA of Lisbon, Lisbon, Portugal; 10 Gravitational Physiology and Medicine Research Unit, Division of Physiology and Pathophysiology, Otto Loewi Research Center of Vascular Biology, Immunity and Inflammation, Medical University of Graz, Graz, Austria

**Keywords:** isolation and confinement, muscle fatigue, muscle strength, SIRIUS-21 analog mission, skeletal muscle function, space flight, stress induced by isolation, network Physiology

## Abstract

**Introduction:**

There is growing interest in exploring outer space to expand scientific knowledge and access valuable material resources. However, progress is limited by the harsh space environment, including microgravity, ionizing radiation, isolation and confinement (IC), disrupted circadian rhythms, and nutritional challenges. These stressors contribute to health problems across multiple organ systems, particularly the musculoskeletal, immune, renal/urinary, and cardiovascular systems. Although we have extensive insight into skeletal muscle pathophysiological changes induced by these spaceflight-related stressors, the molecular mechanisms underlying functional alterations caused by prolonged IC in skeletal muscle remain incompletely understood.

**Methods:**

During the 8-month SIRIUS-21 space analog mission, five participants (3 males, 2 females) underwent plasma and saliva sampling and electromyography (EMG) assessment at five time points: baseline (Ingress), early, mid, and late isolation and confinement (IC), and 14 days post-mission (Egress). Metabolites were analyzed by LC-MS/MS. Longitudinal metabolite changes were assessed using repeated-measures ANOVA with Tukey HSD *post hoc* testing, and direct metabolite-EMG relationships were explored using Spearman rank correlations.

**Results:**

Metabolomic profiling identified 45 skeletal muscle-related metabolites with nominally significant longitudinal changes (p < 0.0001–0.05): 8 were detected in both plasma and saliva, 26 only in plasma, and 11 only in saliva. Direct exploratory Spearman correlations with EMG metrics were identified for a subset of metabolites, including pipecolic acid, phenylacetic acid, L-phenylalanine, L-acetylcarnitine, L-glutamine, and pyruvaldehyde. EMG findings showed a progressive decline in muscle activation and fatigue resistance during prolonged IC, with partial recovery after egress. Exploratory sex-related analyses were interpreted descriptively because the cohort included only two females and three males.

**Conclusion:**

Prolonged IC was accompanied by coordinated changes in muscle function and circulating or salivary metabolites. The identified metabolites represent candidate metabolic signatures associated with muscle dysfunction and require validation in larger independent cohorts before diagnostic or therapeutic utility can be established.

## Introduction

Exploring the outer space has recently gained significant interest due to its multitude of potential benefits, including the possibility of establishing new habitats rich in natural resources. However, the harsh space environment presents major challenges to achieving these goals especially for long-duration human spaceflight. Key environmental stressors include cosmic radiation, microgravity, disrupted circadian rhythms, specialized diets, and psychosocial factors such as isolation and confinement (IC) (Schakman et al., 2013; Belavy et al., 2013; Goswami et al., 2026a; Goswami et al., 2026b). These conditions have substantial impacts on human health, leading to musculoskeletal atrophy, reduced bone density, cardiovascular deconditioning, and immune and metabolic disturbances. Such alterations become more pronounced during long-term missions and manifest across organ-system, cellular, and molecular levels, underscoring the need for expanded large-scale research (Schakman et al., 2013; Belavy et al., 2013; Goswami et al., 2013).

While space medicine research has made significant progress, the specific physiological consequences of stress induced by isolation and confinement remain incompletely understood. Emerging evidence suggests that the pathophysiological changes observed under IC conditions are partly mediated by the activation of stress-responsive neuroendocrine pathways. IC is known to induce characteristic neurohormonal changes, including heightened sympathetic nervous system activity and increased circulating levels of stress hormones such as cortisol, which collectively affect cardiovascular, immune, and metabolic functions (Pagel and Chouker, 1985).

Skeletal muscle, accounting for nearly 40%–50% of total body mass, is profoundly affected during spaceflight due to a combination of microgravity, reduced physical activity, and stress-related factors such as IC (Cacioppo et al., 2015). Among the most well-documented physiological adaptations to microgravity are muscle atrophy, reduced muscle strength, and impaired oxidative metabolism (Cacioppo et al., 2015). These changes predominantly affect postural and lower-limb muscles, which are normally engaged in gravitational resistance on Earth. Although structured in-flight exercise programs represent the most effective countermeasure to mitigate muscle loss, simulated microgravity conditions and ground-based analogs (e.g., bed rest, dry immersion, or isolation chambers) consistently demonstrate that the absence of gravitational loading and physical activity continues to drive skeletal muscle deterioration (Schakman et al., 2013; Cacioppo et al., 2015).

Importantly, while the mechanical and metabolic stressors affecting muscle integrity during spaceflight are well studied, the specific contributions of IC, particularly its neuroendocrine and psychosocial stress components, on skeletal muscle structure and function remain poorly characterized. The potential molecular mechanisms linking chronic stress, altered hypothalamic-pituitary-adrenal (HPA) axis activity, and muscle catabolism in these contexts are still under investigation, highlighting a critical gap in our understanding of human adaptation to prolonged space missions. Previous studies have reported that IC during space analog missions can lead to changes in skeletal muscle function (Gaffney et al., 2017). However, the molecular mechanisms underlying these responses remain incompletely understood. This work builds on our previous findings demonstrating reduced muscle strength and increased muscle fatigue during IC missions (Al Falasi S, et al., 2026, manuscript under review). The primary objective was to identify metabolic signatures in plasma and saliva associated with skeletal muscle function during long-term IC and to explore their relationships with EMG-derived measures of muscle function.

## Materials and methods

### Study design and population

The SIRIUS-21 analog mission was conducted as a collaborative project between Roscosmos, NASA, and the Mohammed Bin Rashid Space Centre (MBRSC, Dubai). The study was performed at the refurbished ground-based isolation facility of the Institute of Biomedical Problems (IBMP), Moscow, Russia, between November 2021 and July 2022. Following a protocol modification due to injury-related early withdrawal of one female crew member, the final cohort comprised five participants (2 females, 3 males). The crew had a mean age of 34.75 ± 5.44 years, height of 170.00 ± 2.00 cm, body weight of 74.50 ± 12.53 kg, and body mass index (BMI) of 25.47 ± 3.94 kg/m^2^. The mission involved 254 consecutive days of isolation and confinement (IC). Physiological and biological measurements were collected at five predefined time points across the study period (Figure 1).

**FIGURE 1:**
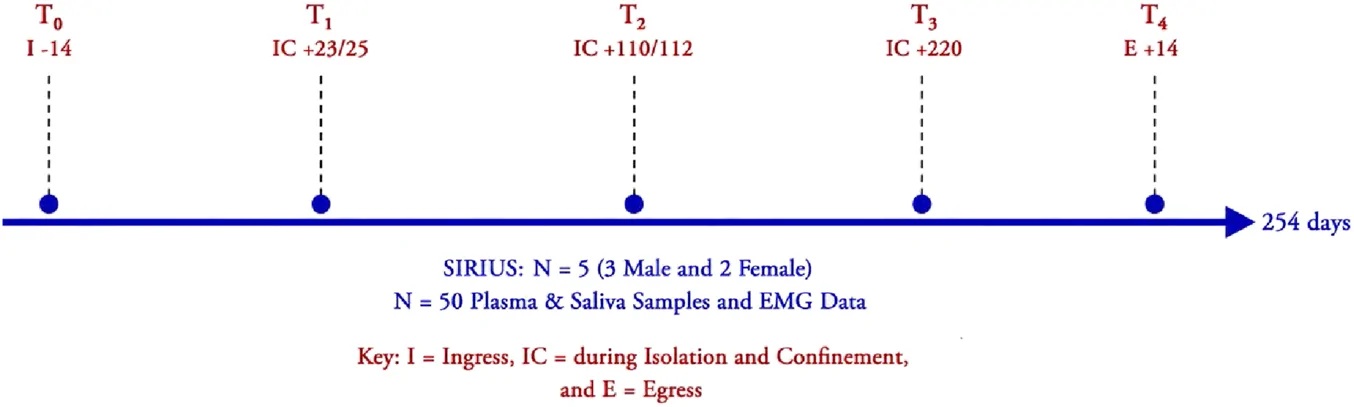
The graph illustrates the five (Goswami et al., 2013) sampling periods during the SIRIUS-21, 254-day Mission for each of the five (Goswami et al., 2013) crew members. T_0_ = Period before IC, T_1_ = 1^st^ sampling period during IC. T_2_ = 2^nd^ sampling period during IC. T_3_ = 3^rd^ sampling period during IC, and T_4_ = sampling period after IC.

### Surface electromyography (sEMG) acquisition and analysis

Surface electromyography (sEMG) was recorded using the Trigno Wireless EMG System (Delsys Inc.) integrated with a PowerLab data acquisition unit (ADInstruments). Six Trigno sensors were placed on the participant’s dominant upper and lower limbs: biceps (UL1), triceps (UL2), forearm flexor region (UL3), quadriceps (LL1), hamstrings (LL2), and calf (LL3). Sensors were paired using the Trigno Control Utility, fitted with adhesive interfaces, and positioned over the muscle belly with fib er-aligned, vertical orientation. The base station was connected to a laptop, and LabChart was launched using a standardized EMG configuration file (Gohel and Mehendale, 2020). No resistance-training countermeasures were implemented outside the study-specific measurement protocol. Participants performed standardized flexion–extension movements of the elbow, hand, hip, and foot (five repetitions each) under both non-resistive and resistive conditions, followed by a 60-s maximal voluntary contraction using a handgrip dynamometer. All signals were recorded in real time through LabChart.

### Statistical analysis

sEMG mean frequency (sEMGMNF) was analyzed using a mixed-effects model with participant as a random effect and sampling time (T0-T4), muscle group (UL1-UL3, LL1-LL3), and contraction condition (resistive vs. non-resistive) as fixed effects. Interaction terms among time, muscle group, and contraction condition were examined to assess whether temporal responses differed by muscle or condition. Model estimates are reported with 95% confidence intervals where available. For the exploratory metabolite-EMG analysis, each Spearman correlation was calculated from five time-point-level summary values (T0-T4; n = 5 observations per correlation), rather than from pooled participant-level repeated observations. Spearman rank correlation coefficients (ρ) were therefore used descriptively to summarize the direction and magnitude of monotonic associations. Because the effective sample size was only five and multiple exploratory correlations were examined without multiplicity correction, emphasis is placed on the direction and magnitude of ρ rather than nominal statistical significance. Nominal two-sided p-values are provided in Tables 1–3 for transparency and should be interpreted very cautiously. Robust response screening using Huber M-estimation in JMP 17 (SAS Institute) was a separate analysis; FDR correction was applied within that response-screening family of tests, with p_FDR <0.05 used for that analysis only.

**TABLE 1:** Eight of the 45 skeletal muscle-related metabolites showing nominally significant longitudinal changes in the mixed cohort (p < 0.0001–0.05) were detected in both plasma (P) and saliva (S). Direct metabolite–EMG relationships were explored using Spearman’s rank correlation (ρ) based on five time-point-level observations (T0-T4; n = 5 per correlation); nominal p-values are shown descriptively, without multiplicity correction. Metabolites with identified direct EMG correlations are highlighted in light green. R, resistive; NR, non-resistive; UL, upper limb; LL, lower limb.

No.	Metabolite	Biofluid	Longitudinal p-value	Functional status	Tukey HSD differences	Direct EMG metric(s)	Spearman rho	Nominal Spearman p (descriptive)
1	Pipecolic acid	P	<0.0001	Direct	T0 & T1-4, T1,3 & T4	LL1-R	0.9	0.0374
1	Pipecolic acid	S	<0.0001	Direct	T0-3 & T4	LL1-R	0.9	0.0374
2	Uracil	P	<0.0001	Direct/Indirect	T0 & T1-4 T1 & T4	None identified	None identified	None identified
2	Uracil	S	<0.0371	Direct/Indirect	T0 & T4	None identified	None identified	None identified
3	Succinylacetone	P	0.0009	Direct	T0 & T2-4	LL3-NR UL3-NR UL-NR	−0.9 0.9 0.9	0.0374 0.0374 0.0374
3	Succinylacetone	S	0.0067	Direct	T0-3 & T4	LL3-NR UL3-NR UL-NR	−0.9 0.9 0.9	0.0374 0.0374 0.0374
4	Sphingosine	P	<0.0001	Direct/Indirect	T0 & T1-4	None identified	None identified	None identified
4	Sphingosine	S	0.0081	Direct/Indirect	T0 & T1-4	None identified	None identified	None identified
5	L-Phenylalanine	P	0.0005	Direct/indirect	T0-1 & T3-4	LL3-R	−0.9	0.0374
5	L-Phenylalanine	S	0.0082	Direct/indirect	T0-3 & T4	LL3-R	−0.9	0.0374
6	Phenylacetic acid	P	0.0308	Direct	T0-1 & T3-4	LL-R LL2-R All -R	−1 −0.9 −0.9	<0.0001 0.0374 0.0374
6	Phenylacetic acid	S	0.0004	Direct	T0-1 & T3-4	LL-R LL2-R All -R	−1 −0.9 −0.9	<0.0001 0.0374 0.0374
7	Niacinamide	P	0.0288	Direct	T1 & T4	None identified	None identified	None identified
7	Niacinamide	S	0.0024	Direct	T0 & T1-4	None identified	None identified	None identified
8	2-Phenylbutyric acid	P	0.0081	Direct	T0-1 & T3	LL1-R	0.9	0.0374
8	2-Phenylbutyric acid	S	0.0047	Direct	T1,3 & T4	LL1-R	0.9	0.0374

Study sample: n = 5 participants (2 females, 3 males), assessed at five longitudinal time points (T0 pre-isolation; T1-T3 during isolation; T4 post-isolation). Longitudinal p-values are nominal p-values from the repeated-measures metabolite analysis. ρ = Spearman rank correlation coefficient. Nominal two-sided Spearman p-values are shown as reported in the available correlation output; no FDR-adjusted Spearman p-values were available. For Table 1 metabolites detected in both biofluids, the available Spearman output did not identify biofluid for individual correlation rows; therefore, the same correlation cannot be assigned specifically to plasma versus saliva. R = resistive; NR, non-resistive; UL, upper limb; LL, lower limb; All = grouped EMG, metric. “None identified” indicates that no direct metabolite-EMG, Spearman association was identified in the available analysis. Spearman correlations were calculated from five time-point-level summary values (T0-T4; n = 5 observations per correlation), not from pooled participant-level repeated measurements. Given this very small effective sample size and the absence of multiplicity correction, ρ is presented primarily to show association direction and magnitude; nominal p-values are descriptive and should be interpreted cautiously.

**TABLE 2:** Twenty-six of the 45 skeletal muscle-related metabolites showing nominally significant longitudinal changes in the mixed cohort (p < 0.0001–0.05) were detected in plasma (P) only. Direct metabolite–EMG relationships were explored using Spearman’s rank correlation (ρ) based on five time-point-level observations (T0-T4; n = 5 per correlation); nominal p-values are shown descriptively, without multiplicity correction. Metabolites with identified direct EMG correlations are highlighted in light green. R, resistive; NR, non-resistive; UL, upper limb; LL, lower limb.

No.	Metabolite	Biofluid	Longitudinal p-value	Functional status	Tukey HSD differences	Direct EMG metric(s)	Spearman rho	Nominal Spearman p (descriptive)
1	N-Acetyl-L-alanine	P	<0.0001	Direct	T0 & T1-4	LL3-R	1	<0.0001
2	Guanidine	P	0.0003	Direct	T0 & T1,4 T1 & T4 T2,3 & T4	LL-NR LL2-NR LL2-R All -R	−1 −0.9 −0.9 −0.9	<0.0001 0.0374 0.0374 0.0374
3	Oxoglutaric acid	P	0.0010	Direct	T0,1 & T4	LL1-NR	−0.9	0.0374
4	Glutathione	P	0.0011	Direct	T0 & T1-4	None identified	None identified	None identified
5	L-Methionine	P	0.0018	Direct	T0 & T2-4	None identified	None identified	None identified
6	L-Arginine	P	0.0021	Direct	T0,1,3 & T4	LL1-NR LL -R	−0.9 −0.9	0.0374 0.0374
7	L-Histidine	P	0.0028	Direct	T0-1, 3 & T4	None identified	None identified	None identified
8	Uridine	P	0.0029	Direct	T0-1 & T4	None identified	None identified	None identified
9	L-Acetylcarnitine	P	0.0032	Direct	T0,1,3 & T4	UL2-NR UL-NR	−1 −0.9	<0.0001 0.0374
10	(R)-Lipoic acid	P	0.0040	Direct	T0,1 & T4	LL2-R LL3-NR UL3-NR LL-NR LL -R	−1 −0.9 0.9 −0.9 −0.9	<0.0001 0.0374 0.0374 0.0374 0.0374
11	Theobromine	P	0.0057	Direct	T0 & T1-2	None identified	None identified	None identified
12	1-Methyladenosine	P	0.0068	Direct	T0 & T2,4	LL3-NR UL3-NR LL2-R	−1 1 −0.9	<0.0001 <0.0001 0.0374
13	m-Coumaric acid	P	0.0085	Direct	T0 & T2,4	None identified	None identified	None identified
14	Deoxycholic acid glycine conjugate	P	0.0086	Direct	T0 & T1-4	LL1-NR LL -R	0.9 0.9	0.0374 0.0374
15	Citric acid	P	0.0101	Direct	T0-2 & T4	LL-NR LL2-NR LL2-R All -R	−1 −0.9 −0.9 −0.9	<0.0001 0.0374 0.0374 0.0374
16	Stearic acid	P	0.0107	Direct	T0 & T3-4	UL2-NR LL2-R	−0.9 0.9	0.0374 0.0374
17	Asymmetric dimethylarginine (ADMA)	P	0.0221	Direct	T0,1 & T4	None identified	None identified	None identified
18	Hydrocinnamic acid	P	0.0234	Direct/Indirect	T1 & T3	None identified	None identified	None identified
19	Trimethylamine	P	0.0249	Direct/Indirect	No HSD Differences	LL3-R	0.9	0.0374
20	Ethanolamine	P	0.0274	Direct	T1 & T3	All-NR	0.9	0.0374
21	L-Cystine	P	0.0313	Direct	T1 & T3	LL3-R	−0.9	0.0374
22	L-Leucine	P	0.0303	Direct	T0 & T4	None identified	None identified	None identified
23	3-Methylhistidine	P	0.0398	Direct	T0 & T2	All-NR	−0.9	0.0374
24	L-Aspartic acid	P	0.0415	Direct	T0 & T1	None identified	None identified	None identified
25	Stachyose	P	0.0409	Direct/Indirect	No HSD Differences	All-NR	0.9	0.0374
26	Cortisol	P	0.0497	Direct	T0 & T1	None identified	None identified	None identified

Study sample: n = 5 participants (2 females, 3 males), assessed at five longitudinal time points: T0 pre-isolation, T1-T3 during isolation, and T4 post-isolation. ρ = Spearman rank correlation coefficient. Nominal two-sided Spearman p-values are shown exactly as reported in the appendix. No FDR-adjusted Spearman p-value is reported. R = resistive; NR, non-resistive; UL, upper limb; LL, lower limb; All = grouped EMG, metric. Spearman correlations were calculated from five time-point-level summary values (T0-T4; n = 5 observations per correlation), not from pooled participant-level repeated measurements. Given this very small effective sample size and the absence of multiplicity correction, ρ is presented primarily to show association direction and magnitude; nominal p-values are descriptive and should be interpreted cautiously.

**TABLE 3:** Eleven of the 45 skeletal muscle-related metabolites showing nominally significant longitudinal changes in the mixed cohort (p < 0.0001–0.05) were detected in saliva (S) only. Direct metabolite–EMG relationships were explored using Spearman’s rank correlation (ρ) based on five time-point-level observations (T0-T4; n = 5 per correlation); nominal p-values are shown descriptively, without multiplicity correction. Metabolites with identified direct EMG correlations are highlighted in light green. R, resistive; NR, non-resistive; UL, upper limb; LL, lower limb.

No.	Metabolite	Biofluid	Longitudinal p-value	Functional status	Tukey HSD differences	Direct EMG metric(s)	Spearman rho	Nominal Spearman p (descriptive)
1	5-Dodecenoic acid	S	0.0001	Direct	T0-1 & T3-4 T2 & T4	None identified	None identified	None identified
2	2-Ethyl-2-hydroxybutyric acid	S	0.0003	Direct	T0-3 & T4	None identified	None identified	None identified
3	Pyridoxal	S	0.0003	Direct	T0 & T1-4	None identified	None identified	None identified
4	L-Glutamine	S	0.0070	Direct	T0,2 & T4	UL2-NR UL-NR	1 0.9	<0.0001 0.0374
5	7-Ketocholesterol	S	0.0008	Direct	T0 & T3-4, T1 & T2-4	None identified	None identified	None identified
6	Pyruvaldehyde	S	0.0028	Direct	T0 & T2-4	All-NR LL2-NR All -R	1 0.9 0.9	<0.0001 0.0374 0.0374
7	Adenosine	S	0.0041	Direct	T0 & T1,3 T1 & T4	All-NR	0.9	0.0374
8	Sucrose	S	0.0062	Direct	T0-3 & T4	None identified	None identified	None identified
9	Etiocholanolone	S	0.0101	Direct/Indirect	T1 & T3-4	None identified	None identified	None identified
10	Isovalerylcarnitine	S	0.0234	Direct	T0 & T4	All-NR UL2-R UL -R	0.9 0.9 0.9	0.0374 0.0374 0.0374
11	2-Ketohexanoic acid	S	0.0458	Direct	No HSD Differences	All-NR	0.9	0.0374

Study sample: n = 5 participants (2 females, 3 males), assessed at five longitudinal time points (T0 pre-isolation; T1–T3 during isolation; T4 post-isolation). Longitudinal p-values are nominal p-values from the repeated-measures metabolite analysis. ρ = Spearman rank correlation coefficient. Nominal two-sided Spearman p-values are shown as reported in the available correlation output; no FDR-adjusted Spearman p-values were available. R = resistive; NR, non-resistive; UL, upper limb; LL, lower limb; All = grouped EMG, metric. “None identified” indicates that no direct metabolite–EMG, Spearman association was identified in the available analysis. Spearman correlations were calculated from five time-point-level summary values (T0-T4; n = 5 observations per correlation), not from pooled participant-level repeated measurements. Given this very small effective sample size and the absence of multiplicity correction, ρ is presented primarily to show association direction and magnitude; nominal p-values are descriptive and should be interpreted cautiously.

## Metabolomics from plasma and saliva samples

### Plasma and saliva samples preparation and metabolite extraction

Blood and saliva samples were collected five times during the SIRIUS-21 mission via salivary sampling and phlebotomy. A volume of 10 mL of whole blood was collected from each participant using EDTA tubes. The collected blood was gently inverted 8-10 times to ensure proper mixing, after which the samples were centrifuged at 4200 RPM for 10 min. This centrifugation process resulted in the separation of 4 mL of plasma, which was then transferred to 2 mL tubes. The plasma samples were immediately placed in a −80 °C freezer for storage. As previously described (Banimfreg et al., 2022), for metabolite extraction plasma samples were processed by adding cold methanol (3:1 v/v), followed by vortexing and incubation at −20 °C for two hours. After centrifugation at 20,817 × g for 15 min at 4 °C, the supernatant was collected and dried using a SpeedVac at 30 °C–40 °C. The dried extracts were either stored at −80 °C or reconstituted in 0.1% formic acid (3:1 v/v) before LC-MS/MS analysis. The prepared samples were then placed in the autosampler for analysis. The mixed-cohort data were then analyzed statistically, with metabolites showing nominally significant longitudinal changes ranging from p < 0.0001 to p < 0.05.

Saliva samples were collected from each participant around each blood-draw time point. To minimize circadian and feeding effects, participants collected samples in the morning immediately after waking and before eating or brushing their teeth. Approximately 1–2 mL of saliva was collected by passive drool into a sterile, low-binding, screw-cap tube and frozen at −80 °C until analysis. For metabolite extraction, saliva samples were processed by adding cold methanol at a 3:1 (v/v) methanol-to-sample ratio, followed by vortex mixing and incubation at −20 °C for 2 h. The samples were then centrifuged at 20,817 × g for 15 min at 4 °C, and the resulting supernatants were collected and dried using a SpeedVac at 30 °C–40 °C. The dried extracts were either stored at −80 °C until further analysis or reconstituted in 0.1% formic acid at a 3:1 (v/v) ratio prior to LC-MS/MS analysis. The reconstituted samples were transferred to autosampler vials and placed in the autosampler for LC-MS/MS analysis.

### LC-MS/MS-based metabolomics analysis

Metabolomic profiling was conducted using a timsTOF Pro mass spectrometer (Bruker, Germany) coupled with an ELUTE UHPLC system and controlled by Bruker Compass HyStar 5.0 and MetaboScape 4.0 software. The system utilized a trapped ion mobility quadrupole time-of-flight (timsTOF) mass analyzer equipped with an electrospray ionization (ESI) source operated in positive auto MS/MS scan mode across a mass range of m/z 20–1300 (Ibrahim et al., 2024).

Metabolite separation was achieved using a HAMILTON® Intensity Solo 2 C18 column (100 μm × 2.1 mm, 1.8 μm particle size). The mobile phases consisted of solvent A (water +0.1% formic acid) and solvent B (acetonitrile +0.1% formic acid), delivered in gradient mode at a flow rate of 0.25–0.35 mL/min over 30 min. The column oven was maintained at 35 °C, and the autosampler was set to 8 °C. The injection volume was 10 μL, and external calibration was performed using sodium formate. ESI parameters included a drying gas flow of 10 L/min, drying temperature of 220 °C, capillary voltage of 4500 V, nebulizer pressure of 2.2 bar, collision energy of 7 eV, and end plate offset of 500 V. The total ion chromatograms (TIC) and metabolite fragmentation patterns were analyzed using the Human Metabolome Database (HMDB) mass spectral library. All samples were analyzed in technical duplicates.

### Metabolomics quality control and preprocessing

A quality control (QC) sample was prepared by pooling equal volumes of each sample to evaluate the reproducibility of the analytical procedure. The samples were analyzed in a randomized sequence. The analytical sequence began with five injections of solvent A (0.1% formic acid in deionized water) for system equilibration, followed by five injections of the pooled QC sample. Subsequently, one pooled QC sample was injected after every 9–10 study samples to monitor analytical reproducibility throughout the analytical sequence. The pooled QC injections were also used to monitor analytical stability and potential drift. No substantial analytical drift affecting the retained features was observed. Features showing an RSD >30% across QC samples were excluded from subsequent analysis.

Data processing was performed using MetaboScape 4.0 software (Bruker Daltonics). Feature detection and bucketing were performed using the T-ReX 3D workflow. The parameters for molecular feature detection were set to a minimum intensity threshold of 1,000 counts and a minimum peak length of 7 spectra, with peak intensity used for feature quantitation. Mass recalibration was performed within a retention time range of 0–0.3 min. During the initial feature detection step, only features present in at least 70% of the samples in at least one experimental group were retained. The MS/MS import method was set to averaging. For data bucketing, the retention time range was 0.3–25 min and the mass range was 50–1,300 m/z.

Following generation of the bucket table, metabolite annotation was performed by matching MS/MS spectra against spectral libraries using the MetaboScape workflow, incorporating the Bruker MetaboBASE Personal Library 3.0 and Bruker HMDB Metabolite Library 2.0. For features with multiple annotations, the annotation with the highest annotation quality (AQ) score was retained based on MS/MS spectral matching, m/z accuracy, and mSigma values. Metabolite annotations were accepted based on an m/z mass error of <5 ppm and an MS/MS spectral matching score >400, together with the isotope pattern quality assessed by the mSigma value.

Following annotation, technical replicates were averaged to obtain a single value per sample; when one replicate was missing, the available value was retained. Features with a relative standard deviation (RSD) > 30% in QC samples were excluded, and metabolites detected in at least 70% of samples within at least one experimental group were retained. Remaining missing values were imputed using a half-minimum approach. The data were subsequently normalized using median normalization, followed by log2 transformation prior to statistical analysis.

Metabolomics Data Analysis A repeated-measures analysis of variance (ANOVA) model was used to test longitudinal differences in metabolite abundance, with participant treated as a random effect and time (T0-T4) as a fixed effect. Tukey-Kramer HSD *post hoc* tests compared pairs of sampling time points. Residual-by-predicted plots and residual distributions were examined to assess approximate normality, centering around zero, and constant variance. Skewed outcomes were log-transformed and model diagnostics were reassessed after transformation. Potentially influential observations were evaluated by comparing models with and without the observation; because no prespecified numerical outlier threshold was documented in the original analysis, this sensitivity procedure is now stated explicitly rather than implying a formal outlier rule. The 45 metabolites in Tables 1–3 were selected on the basis of nominal longitudinal ANOVA p < 0.05 together with skeletal-muscle relevance; they should therefore be interpreted as nominally significant exploratory findings rather than FDR-significant discoveries. Spearman rho and nominal p-values for direct metabolite-EMG associations are also reported in Tables 1–3. FDR-adjusted values reported for other analyses (including robust response screening) refer only to their respective prespecified test families and are not FDR-adjusted Spearman p-values. Given only five independent participants, sex comparisons are presented as descriptive/exploratory observations rather than confirmatory between-sex inference.

## Results

The present study is a continuation of our recent study (Elmoselhi et al., 2024), and included five adult participants (three males and two females) using plasma and saliva metabolites and EMG data taken during SIRIUS-21 (Figure 1) to determine metabolomic effects concerning skeletal muscle function due to stress induced by IC.

### Electromyographic (EMG) adaptations during isolation and confinement

Mean EMG frequency differed across muscle groups during the 254-day isolation period (Figure 2). The mixed-effects analysis examined muscle group x time and contraction-condition effects. Mean EMG frequency was modestly higher during resistive than non-resistive contractions (LS mean approximately 80 Hz, 95% CI 74.616–85.983 vs. approximately 77 Hz, 95% CI 71.118–82.484). Because both conditions were performed only within the EMG assessment protocol, this difference reflects acute task conditions and should not be interpreted as evidence that resistance exercise acted as a protective countermeasure during the mission.

**FIGURE 2:**
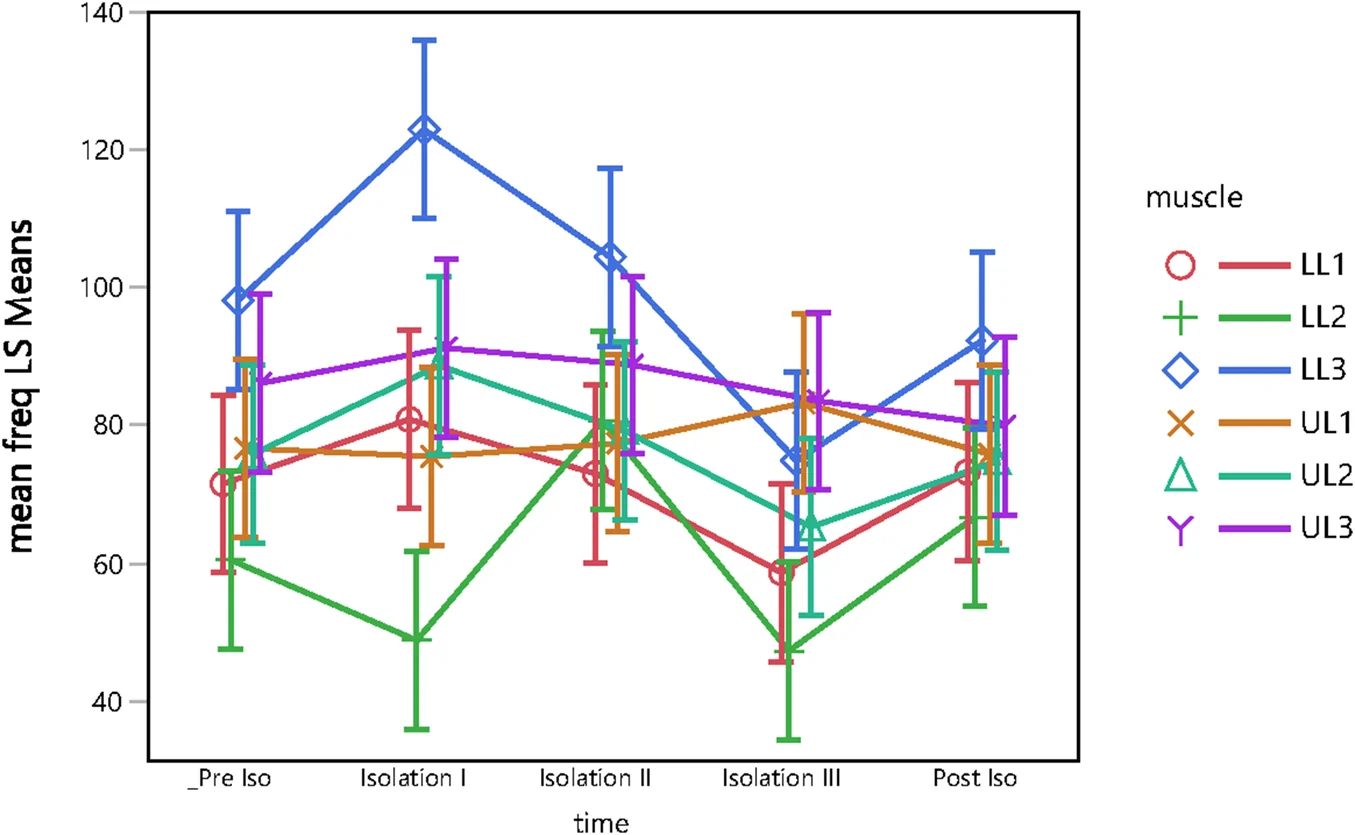
Tukey HSD least squares mean plots (df = 5) are shown for the muscle × time interaction, with a standard error of 2.901. The 95% confidence intervals were 74.616–85.983 for resistive conditions and 71.118–82.484 for non-resistive conditions. Trigno sensors were placed on the participant’s dominant upper and lower limbs: biceps (UL1), triceps (UL2), forearm flexor region (UL3), quadriceps (LL1), hamstrings (LL2), and calf (LL3).

Lower limb muscles (LL1- LL3) demonstrated heterogeneous responses, with calf muscles (LL3) exhibiting relatively higher activation frequencies, whereas hamstrings (LL2) showed consistently lower values. Upper limb muscles (UL1- UL3) displayed intermediate responses, with forearm flexors (UL3) generally maintaining higher frequencies compared to biceps (UL1) and triceps (UL2). These findings indicate muscle-specific susceptibility to prolonged confinement.

Temporally, EMG mean frequency remained relatively stable during early and mid-isolation, followed by a decline during late isolation. Post-isolation measurements demonstrated partial, but incomplete, recovery toward baseline levels. This pattern suggests an initial adaptive phase followed by progressive neuromuscular deconditioning with prolonged confinement. EMG frequencies were modestly higher during resistive contractions than during non-resistive contractions. As these conditions were performed only as part of the EMG assessment protocol, this observation does not establish a countermeasure effect but may provide a rationale for future studies investigating resistance exercise as a potential strategy to mitigate confinement-related neuromuscular deconditioning. Muscles subjected to non-resistive activity exhibited greater reductions over time, particularly in lower limb groups. Assessment of sustained contraction capacity (60-s maximal effort) revealed a gradual reduction in contraction potential across isolation, with the lowest values observed during late confinement. Although partial recovery was evident post-isolation, baseline endurance capacity was not fully restored. Overall, prolonged isolation was associated with progressive reductions in muscle activation and fatigue resistance, providing functional context for the metabolomic alterations described below.

Metabolites Related to EMG in Biological Samples: Of the 295 grouped metabolites analyzed, 45 skeletal muscle function-associated metabolites showed nominally significant longitudinal changes (p < 0.0001–0.05). These comprised 8 detected in both plasma and saliva, 26 detected only in plasma, and 11 detected only in saliva (Tables 1–3). Direct metabolite-EMG relationships were assessed separately using exploratory Spearman rank correlations calculated from five time-point-level summary observations (T0-T4) per correlation. The corresponding ρ values and nominal p-values are reported in Tables 1–3; given n = 5 and the absence of multiplicity correction, interpretation emphasizes the direction and magnitude of ρ rather than nominal significance.

### Metabolites detected in both plasma and saliva samples

Among the eight metabolites found in both plasma and saliva shown in Table 1, exploratory Spearman analyses identified large-magnitude positive or negative associations (|ρ| = 0.9–1.0) between several metabolites and specific EMG metrics based on five time-point-level observations. These included pipecolic acid, succinylacetone, L-phenylalanine, phenylacetic acid, and 2-phenylbutyric acid. The nominal p-values are shown descriptively in Table 1 and should not be interpreted as confirmatory evidence because n = 5 and no multiplicity correction was applied. In contrast, no direct metabolite-EMG Spearman association was identified in the available analysis for Uracil, Sphingosine, and Niacinamide. Overall, these metabolites are associated with biological processes relevant to skeletal muscle function, including energy metabolism through mitochondrial function, glycolysis and fatty acid oxidation, signal transduction, fatigue resistance, and muscle recovery (Scalabrin et al., 2025; Loffler et al., 2005). Some metabolites, including Uracil, Sphingosine, and L-Phenylalanine, may also influence skeletal muscle indirectly through processes involving RNA and protein synthesis, inflammatory regulation, and neurotransmitter function (McNurlan et al., 1994; Caso et al., 2002; Mitchell et al., 2016; Li et al., 2024; Nikolova-Karakashian and Reid, 2011).

Metabolites Detected Only in Plasma Samples: Of the 26 plasma-only metabolites shown in Table 2, several have established biological roles relevant to muscle metabolism, contractility, recovery, or systemic stress. Direct metabolite-EMG Spearman correlations were identified for a subset, including N-acetyl-L-alanine, guanidine, oxoglutaric acid, L-arginine, L-acetylcarnitine (R)-lipoic acid, 1-methyladenosine, deoxycholic acid glycine conjugate, citric acid, stearic acid, trimethylamine, ethanolamine, L-cystine, 3-methylhistidine, and stachyose. For metabolites without an identified direct Spearman association, temporal changes should not be described as correlations with EMG.

Metabolites Detected Only in Saliva Samples: Eleven metabolites were unique to saliva (Table 3). Direct exploratory Spearman correlations with EMG metrics were identified for L-glutamine, pyruvaldehyde, adenosine, isovalerylcarnitine, and 2-ketohexanoic acid. For the remaining saliva-only metabolites, no direct metabolite-EMG Spearman correlation was identified in the available analysis, and their longitudinal changes are therefore described separately from EMG correlation findings.

### Time point dynamics

Tukey HSD analysis revealed that many metabolite concentrations changed significantly at T2 and T3, coinciding with the sharp decline in EMG frequency and muscle contraction potential. Notably, decreased energy-related metabolites (e.g., niacinamide, L-acetylcarnitine) were observed during T2–T3; Elevated stress-related or catabolic indicators (e.g., cortisol, ADMA) peaked in late isolation; Partial recovery was evident post-isolation (T4), though several metabolites (e.g., glutathione, L-leucine, 3-methylhistidine) remained altered, suggesting incomplete metabolic recovery.

### Sex-related differences in plasma metabolites

Exploratory analysis identified nominal sex-related differences in two plasma metabolites, pantothenic acid (vitamin B5) and stachyose, during the 8-month isolation and confinement study (Table 4). After FDR correction, only stachyose remained significant (p_FDR = 0.02052), whereas pantothenic acid did not (p_FDR = 0.12531). Given the very small number of independent participants (2 females and 3 males), these findings are considered descriptive and hypothesis-generating and do not support confirmatory sex-specific inference or conclusions regarding sex-specific metabolic advantages (Tornoposky et al, 2008).

**TABLE 4:** Exploratory analysis of sex-related differences in plasma metabolites. Nominal and false discovery rate (FDR)-adjusted p-values are shown. Given the small cohort (2 females and 3 males), these findings are considered descriptive and hypothesis-generating. Pantothenic acid showed a nominal sex-related difference (p = 0.0418) but did not remain significant after FDR correction (pFDR = 0.12531), whereas stachyose remained significant after FDR correction (pFDR = 0.02052).

No.	Metabolite	Nominal p-value	FDR-adjusted p-value	FDR <0.05	Direction of nominal difference
1	Pantothenic acid (Vit. B5)	0.0418	0.12531	No	Female > Male
2	Stachyose	0.0268	0.02052	Yes	Female > Male

This sex-related analysis is exploratory because the cohort included only five independent participants (2 females, 3 males). Nominal p-values and FDR-adjusted p-values are shown separately. Only stachyose remained significant after FDR, correction (FDR-adjusted p < 0.05); pantothenic acid showed only a nominal sex-related difference.

### Functional pathway analysis of skeletal muscle-related metabolites

Nineteen metabolites remained significant after FDR correction (FDR-adjusted p < 0.05) in the robust response-screening analysis and are presented by functional category according to their relevance to skeletal muscle activity and EMG-associated changes (Table 5).

**TABLE 5:** Functional analysis of 19 metabolites that remained significant after false discovery rate correction (FDR-adjusted p < 0.05), grouped according to metabolic pathways relevant to skeletal muscle function and EMG-associated changes.

No.	Metabolite	Nominal p-value	FDR-adjusted p-value	References & Links
1	Dihydrothymine	<0.0001	0.00009	HMDB0000079 TB5 -(1) Wilkinson, D. J.; Rodriguez-Blanco, G., et al.
2	Thymine	0.0008	0.00169	HMDB0000262 TB5 -(2) Yi, X.; Yang, Y.; Li, T.; Li, M.; Yao, T.; Hu, G., et al.
3	L-Histidine	0.0015	0.00291	HMDB0000177 TB5 -(3) Physiology and Pathophysiology of Carnosine
4	N-Acetyl-L-alanine	0.002	0.00198	TB5 -(4) Lyu, Q.; Zhang, L.; Ding, Y.;
5	Allatonic acid	0.0049	0.00973	HMDB0001209
6	6-Methyladenine	0.0069	0.01389	HMDB0002099 TB5 -(5) Li, J.; Pei, Y.; Zhou, R
7	L-Glutamine	0.0073	0.01464	HMDB0000641 TB5 -(6) Tajari, S. N.; Rezaee, M.; Gheidi, N
8	Pipecolic Acid	0.0089	0.00894	HMDB0000070 TB5 -(7) Sato, T.; Ito,Yoshiaki; and Nagasawa, T
9	Thymidine	0.0092	0.01842	HMDB0000273 TB5 -(8) Sommerland, H.; Ullman, M.; Jennische, E., et al.
10	Guanidine	0.0105	0.01053	HMDB0001842 TB5- (9) Ferrari, R.; Rodrigues-Simioni, L.; da Cruz Höfling, M. A., et al.
11	(R)-Lipoic Acid	0.0131	0.01312	TB5 -(10) Isenmann, E.; Trittel, L.;
12	5-Hydroxy-L-Tryptophan	0.015	0.02653	TB5 -(11) Warden, S. J.; Haney, E. M
13	Uracil	0.0164	0.0164	TB5 -(12) Alsøe, L.; Sarno, A.; Carracedo, S.;
14	5-Hydroxytryptophol	0.0194	0.01943	TB5 -(13) Lands, L. C.; Grey, V. L.; Smountas, A. A
15	L-Cystine	0.0201	0.03133	​
16	17a-Estradiol	0.024	0.0316	TB5 -(14) Bubak, M. P.; Mann, S. N.;
17	7-Methylguanine	0.0343	0.03432	TB5 -(15) Tan, B. H.; Bencsath, F. A.;
18	Benzoic acid	0.0382	0.03818	TB5 -(16) Moffett, R. B.; Tang, A. H
19	N6-Acetyl-L-lysine	0.0404	0.04038	TB5 -(18) Tsuda, M.; Fukushima, A.;

Nineteen metabolites met the FDR-adjusted significance criterion (FDR-adjusted p < 0.05). Phenylephrine, although nominally significant (p = 0.0397), was removed from the FDR-significant set because its FDR-adjusted p-value was 0.0794. Nominal and FDR-adjusted p-values are reported separately.

#### DNA damage and nucleotide turnover

Several nucleobase-related metabolites were elevated, suggesting alterations in nucleotide turnover and potential DNA damage and repair processes. Dihydrothymine (p < 0.0001) and uracil (p = 0.0164) have been associated with DNA damage and repair pathways, which may be altered in aging muscle (Fang et al., 2023; Wi et al., 2020). Elevated thymidine (p = 0.0092) may reflect increased nucleotide turnover or DNA synthesis associated with satellite cell-mediated muscle regeneration (Tatsumi and Allen, 2004). 6-Methyladenine (p = 0.0069) and 7-methylguanine (p = 0.0343) are associated with nucleic acid methylation and may also reflect oxidative stress-related nucleic acid modifications (Shibutani et al., 1991).

#### Amino acid and protein metabolism

Altered amino acid metabolism was evidenced by increased L-histidine (p = 0.0015), a precursor of carnosine, which is important for muscle pH buffering. L-glutamine (p = 0.0073) and pipecolic acid (p = 0.0089) are involved in muscle repair and anabolic signaling (Legault et al., 2015; Zhao et al., 2018). N-acetyl-L-alanine (p = 0.0020) and N6-acetyl-L-lysine (p = 0.0404) are markers of protein turnover and acetylation status, with implications for mitochondrial efficiency (MacIver et al., 2013; Kendrick et al., 2011; Murgia et al, 2024).

#### Redox balance and inflammation

Key antioxidants and redox-regulating metabolites were increased (R)-Lipoic acid (p = 0.0131) is a mitochondrial antioxidant that reduces oxidative stress in active muscle (Packer and Cadenas, 2011). L-cystine (p = 0.0201) supports glutathione production and muscle fatigue resistance (Lin et al., 2026; Margaritelis et al., 2021). 17α-estradiol (p = 0.0240) exhibits anti-inflammatory properties in muscle under metabolic stress (Steyn et al., 2018). Allantoic acid (p = 0.0049) indicates purine turnover under oxidative load (Tsahar et al., 2006).

#### Neuromuscular signaling

Several metabolites were linked to neuromuscular transmission. Guanidine (p = 0.0105) enhances acetylcholine release at the neuromuscular junction (Kalia and Swartz, 2011). 5-Hydroxy-L-tryptophan (5-HTP) (p = 0.0150) and 5-hydroxytryptophol (p = 0.0194) reflect serotonin turnover, which may modulate fatigue (Truyens et al., 2022). Benzoic acid (p = 0.0382) may influence muscle excitability and related metabolic responses (Joyner et al., 2015). Phenylephrine was excluded from the FDR-significant set because its FDR-adjusted p-value was 0.0794.

#### Energy and mitochondrial metabolism

Metabolites involved in mitochondrial bioenergetics included thymine (p = 0.0008), a BAIBA precursor known to stimulate fatty acid oxidation (Elmoselhi et al., 2024; Roberts et al., 2014). N6-acetyl-L-lysine may impair β-oxidation efficiency when elevated (Kendrick et al., 2011), suggesting disruption of energy regulation under muscular load.

## Discussion

Our study demonstrates integrated metabolomic alterations in plasma and saliva associated with skeletal muscle strength and fatigue during prolonged isolation and confinement (IC). Muscle activation progressively declined over the 8-month IC period, with only partial recovery post-isolation. The relatively attenuated decline observed during resistive compared with non-resistive contractions may suggest that mechanical loading influences muscle activation under prolonged IC conditions. However, because resistance training was not implemented as a countermeasure during the mission, these findings should not be interpreted as evidence of a protective effect. Further studies incorporating controlled resistance-training interventions are needed to determine whether resistive exercise can mitigate IC-induced muscle deconditioning. In parallel, muscle fatigue increased progressively, reflecting cumulative physiological stress. Metabolomic profiling identified 45 skeletal muscle-related metabolites showing nominally significant longitudinal changes (p < 0.0001–0.05), including 8 detected in both plasma and saliva, 26 in plasma only, and 11 in saliva only. Exploratory Spearman analyses based on five time-point-level observations identified large-magnitude associations for a subset of these metabolites; these coefficients are descriptive and not confirmatory because of the very small effective sample size and absence of multiplicity correction. Other metabolites showed longitudinal changes without an identified direct EMG association. Exploratory sex-related findings were interpreted descriptively because only five independent participants were available. Collectively, these findings identify candidate metabolic signatures accompanying muscle deconditioning during prolonged IC, while requiring validation in larger independent cohorts.

The findings in this study are consistent with previous studies conducted in spaceflight and space analog environments. In astronauts after long-duration missions aboard the International Space Station (ISS), reductions in muscle cross-sectional area and functional capacity, particularly in antigravity muscles (e.g., quadriceps, gastrocnemius), have been well documented (Fitts et al., 2010; LeBlanc et al., 1995). Similarly, prolonged head-down tilt bed rest, a validated ground-based model of microgravity, has been associated with significant losses in muscle strength and electromyographic activity (Attias et al., 2020; Rittweger et al., 2005). Our results parallel these findings in ISS and head-down tilt bed rest studies, highlighting that even without microgravity, IC restricted mobility, and the hormonal and psychological stressors in IC environments are sufficient to contribute to a measurable muscle deconditioning.

The physiological and molecular mechanisms underlying muscle deconditioning during IC are multifactorial and not completely understood. Physical inactivity results in reductions in mechanical loading, leading to muscle atrophy, decreased neural drive, and metabolic shifts favoring fatigue (Stein and Wade, 2005; Ibrahim et al, 2024). Psychological stress associated with IC may also impair neuromuscular performance, contributing to decreased muscle recruitment and endurance capacity (Convertino, 1996; Demontis et al., 2017).

Because the cohort included only two females and three males, sex-related metabolite patterns were examined descriptively and are considered exploratory. Repeated longitudinal measurements do not increase the number of independent participants available for between-sex inference; therefore, no conclusions regarding sex-specific metabolic advantages or countermeasures are drawn. The dynamic association between metabolomics and EMG measurements in our study reveals that 8 months of IC leads to pronounced metabolic alterations in pathways critical for skeletal muscle function. The fluctuations in metabolite levels across plasma and saliva samples were consistent with the physiological decline observed in EMG mean frequency and contraction potential, particularly during mid to late IC period. Key metabolites such as niacinamide, L-phenylalanine, oxoglutaric acid, and glutathione play direct roles in ATP production, oxidative stress regulation, and muscle recovery, all of which are known to be compromised during spaceflight analog conditions (Fitts et al., 2010; Stein and Wade, 2005; Murgia et al, 2024). The temporal concurrence of changes in EMG indices and these metabolites suggests a possible relationship between metabolic stress and muscle deconditioning under reduced activity and IC, but does not establish a direct association unless supported by the exploratory Spearman analyses. Furthermore, the observed increase in catabolic biomarkers (e.g., 3-methylhistidine, cortisol, ADMA) especially at T2–T3 suggests ongoing protein degradation and vascular impairment, both of which impair endurance and contribute to muscle fatigue (Rittweger et al., 2005; Demontis et al., 2017). Notably, the EMG decline was most significant during T3, coinciding with peak disruptions in amino acid metabolism, mitochondrial energetics, and antioxidant defenses. Some metabolites (e.g., pyridoxal and L-glutamine) were detected only in saliva, supporting further evaluation of saliva as a non-invasive matrix for candidate metabolic signatures during longitudinal muscle monitoring in IC environments.

Although some post-isolation recovery was observed, several markers related to mitochondrial stress and protein turnover (e.g., stearic acid, 7-ketocholesterol, and 2-phenylbutyric acid) remained altered at T4, suggesting incomplete metabolic recovery and supporting the need for longer post-egress follow-up.

Exploratory sex-related analysis identified nominal differences in pantothenic acid and stachyose. After FDR correction, only stachyose remained significant (p_FDR = 0.02052), whereas pantothenic acid did not (p_FDR = 0.12531). Given the very small number of independent participants (2 females and 3 males), these observations are descriptive and hypothesis-generating only and should not be interpreted as evidence of sex-specific metabolic advantages or used to guide sex-specific countermeasures.

The metabolomic findings indicate coordinated changes in pathways related to nucleotide turnover, amino acid metabolism, redox regulation, neurotransmission, and mitochondrial function during prolonged IC. These observations, together with the exploratory EMG correlations, support further evaluation of combined metabolomic and EMG monitoring in larger studies. They do not establish diagnostic performance, therapeutic efficacy, or the effectiveness of any exercise countermeasure. Increased L-histidine, glutamine, and pipecolic acid highlight anabolic activity and recovery processes. These metabolites contribute to carnosine synthesis, muscle repair, and protein synthesis stimulation (Legault et al., 2015; Everaert et al., 2013). N6-acetyl-L-lysine suggests altered mitochondrial protein acetylation, which may impair β-oxidation (Kendrick et al., 2011). Redox metabolites such as lipoic acid, cystine, and 17α-estradiol reflect oxidative stress responses and anti-inflammatory defense. These changes align with known exercise-induced increases in ROS and the muscle’s requirement to maintain redox homeostasis (Steyn et al., 2018). The presence of guanidine, 5-HTP, and benzoic acid supports modulation of neuromuscular junctions and serotonergic pathways. These may influence fatigue perception, muscle excitation, and vascular regulation during exertion (Yadav et al., 2010; Bosutti et al, 2026). Finally, thymine and its link to β-aminoisobutyric acid (BAIBA) production suggest that muscle tissue engages in endocrine-like signaling to promote systemic lipid metabolism, consistent with the role of BAIBA in exercise adaptation (Roberts et al., 2014).

The most notable limitation of our study is the small sample size, which is inherent to prolonged spaceflight analog studies and limits the generalizability of our findings. Accordingly, these results should be interpreted with caution and considered exploratory, warranting validation in larger and more diverse cohorts. In particular, the imbalance between female and male participants limits our ability to draw definitive sex-specific conclusions. In addition, potential confounding factors, including variations in diet and physical activity, may have influenced plasma and salivary metabolite profiles during the prolonged period of isolation and confinement. Although the controlled nature of the analog environment minimizes some sources of variability, the potential contribution of these factors cannot be completely excluded. Future studies with larger, sex-balanced cohorts and more detailed control or assessment of dietary and exercise-related factors will be important to validate and extend the present findings.

## Conclusion

Prolonged isolation and confinement was associated with progressive changes in EMG-derived muscle function and with nominally significant longitudinal changes in 45 skeletal muscle-related metabolites. A subset showed direct exploratory Spearman correlations with EMG metrics, while others showed temporal changes without an identified direct correlation. Because of the small cohort, particularly the limited number of independent participants for sex comparisons, these findings should be considered hypothesis-generating. The identified metabolites are candidate metabolic signatures associated with muscle dysfunction; larger independent studies are required to establish reproducibility and any future diagnostic or therapeutic utility.

## Data Availability

The datasets presented in this study can be found in online repositories. The names of the repository/repositories and accession number(s) can be found below: https://www.metabolomicsworkbench. org/data/DRCCMetadata.php?Mode=Project&ProjectID=PR003094,PR003094. Project ID PR003094. The data can be accessed directly via it's Project DOI: 10.21228/M8RC41. https://doi.org/http://dx.doi.org/10.21228/M8RC41.
